# Supplementary material for: Saccharomyces boulardii reduces food intake and attenuates weight gain in diet-induced obese mice: links to gut microbial, metabolic, and immune remodeling
Source: Front Immunol. 2026 Sep 29;17:1925200. doi: 10.3389/fimmu.2026.1925200 (PMC13658089; doi:10.3389/fimmu.2026.1925200)
Supplement: Supplementary file 1 [file DataSheet1.docx]

**Supplementary Figures**

*Saccharomyces boulardii* reduces food intake and attenuates weight gain in diet-induced obese mice: links to gut microbial, metabolic, and immune remodeling

Authors: Karl Alex Hedin^1,2,3,*^, Troels Holger Vaaben^1,2^, Ditte Olsen Lützhøft^1^, Benjamin A. H. Jensen^4^, Morten Otto Alexander Sommer^1,2,*^

1. ^Novo Nordisk Foundation Center for Biosustainability, Technical University of Denmark, DK-2800, Kongens Lyngby, Denmark^
2. ^The Novo Nordisk Foundation Biotechnology Research Institute for the Green Transition, Technical University of Denmark, DK-2800 Kongens Lyngby, Denmark^
3. ^1stBiome ApS, DK-2200, Copenhagen, Denmark^
4. ^Department of Biomedical Sciences, Faculty of Health and Medical Sciences, University of Copenhagen, DK-2200 Copenhagen, Denmark^

^*Corresponding authors^

^Morten Otto Alexander Sommer (^[^msom@dtu.dk^](mailto:msom@dtu.dk)^)^

^Karl Alex Hedin (^[^alex@1stbiome.com^](mailto:alex@1stbiome.com)^)^

**Supplementary Figure S1. *S. boulardii* increases body-weight-normalized energy expenditure during the dark phase in mice with DIO. (A)** Hourly energy expenditure (EE; kcal/kg/h) over a 2-day period under a 12:12 light–dark cycle in PBS- and *S. boulardii*-treated mice. Shaded areas indicate the dark phase. **(B)** Mean EE during the light and dark phases. Data are mean ± SEM (n = 9 per group). Statistical analyses were performed using a linear mixed-effects model fitted with lmer (REML = FALSE), including Group, Hour, and their interaction as fixed effects and mouse as a random intercept. Fixed effects were evaluated using Type III ANOVA for panel A; and two-tailed Student’s t-test for panel B. **p* < 0.05, ***p* < 0.01.

**Figure S2. Microbial pathway analysis of tryptophan-derived indole metabolism following *S. boulardii* treatment.** Bar plots showing the relative abundance of microbial genes encoding enzymes involved in tryptophan-derived indole metabolism. Enzymatic reactions are annotated with Enzyme Commission (EC) numbers and arranged to reflect the pathway from tryptophan to indole-3-acetaldehyde and indole-3-acetic acid (IAA). Bars represent mean ± SEM with individual mouse-level abundances shown as dots. Group differences were evaluated using two-sided Welch’s t-tests, and multiple comparisons across EC features were corrected using the Benjamini–Hochberg false discovery rate (FDR).

**Figure S3. Cecal metabolomic remodeling following *S. boulardii* treatment in diet-induced obese mice (DIO).** Untargeted metabolomic profiling of cecal samples from mice treated with *S. boulardii* or PBS. Metabolites are shown as log_2_ fold change (*S. boulardii* vs. PBS) across 248 high-confidence compounds and are color-coded by chemical class.

**Figure S4. Targeted quantification of cecal short-chain fatty acids following *S. boulardii* treatment in diet-induced obese (DIO) mice.** Targeted metabolomic profiling of short-chain fatty acids (SCFAs) in cecal samples from mice treated with *S. boulardii* or PBS. Concentrations are shown as absolute values (µmol/g cecal content).
